# Experience of burden of care among adult caregivers of elderly persons in Oyo State, Nigeria: a cross-sectional study

**DOI:** 10.11604/pamj.2022.42.64.32715

**Published:** 2022-05-24

**Authors:** Oludoyinmola Omobolade Ojifinni, Obioma Chukwudi Uchendu

**Affiliations:** 1 School of Clinical Medicine, Faculty of Health Sciences, University of the Witwatersrand, Johannesburg, South Africa,; 2 Department of Community Medicine, College of Medicine, University of Ibadan, Ibadan, Nigeria

**Keywords:** Caregiver, mental health, Zarit burden interview, Nigeria

## Abstract

**Introduction:**

caring for elderly persons is challenging for caregivers due to elderly persons´ increased dependence and reduced physical strength. This study assessed the burden of care experienced by caregivers of elderly persons in family settings.

**Methods:**

this cross-sectional study used a multistage cluster sampling technique to select 1,119 caregivers of elderly persons aged 18-59 years from one rural and one urban local government area in Oyo State, Nigeria. Interviewer-administered questionnaires collected information on caregiving arrangements and burden of care experienced (determined using the modified short version of the Zarit Burden Interview).

**Results:**

caregivers´ mean age was 38.6 ± 8.7 years with 50.2% aged ≥40 years. There were more females (59.8%) than males (40.2%) and 78.4% were married. Only 47.8% were primary caregivers, 54% cared for their parents and 2% cared for non-relatives. Prior to their caregiving, 81% reported good relationships with the elderly. Although 80.3% of the elderly were reported to be fully independent for activities of daily living, 74.0% of the caregivers experienced burden of care with 28.2% reporting severe burden. The odds of burden of care were 10 times higher among rural than urban caregivers (OR=10.09, 95%CI=5.99-17.01); eight times higher among those with poor than those with good mental health status (OR=7.90, 95%CI=4.60-13.57) three times higher among those with dependent than independent elders (OR=2.74, 95%CI=1.68-4.47).

**Conclusion:**

experience of burden of care was high among caregivers in the rural area and those with poor mental health. Community-oriented support including daycare centres and old people´s homes will provide relief to caregivers.

## Introduction

As people age, there is greater physical, mental, and financial vulnerability. Old age is associated with a diminished capacity to work as well as increased vulnerability due to a reduction in physical strength [1]. Old people therefore require assistance with activities they would otherwise have carried out themselves when they were younger [2,3]. The preferred mode of care for older members of a family in both developed and developing countries is care within the family [4-6]. However, while most developed countries have in addition established formal social support systems for older persons in the society including welfare schemes and homes for the care of the elderly, developing nations particularly in Africa mostly have only an informal system of care [7,8]. The elderly in such developing regions often remain within the extended family system where their care and support are the responsibility of the younger members of the family including children and younger siblings [7-10].

Age classification varies between cultures and countries. While the World Health Organization (WHO) and United Nations (UN) set the cutoff age at 60 years, there is variation among countries in both the developed and developing world ranging between 60 and 65 years. This variation is influenced by differences in social stratification, the chronological age of retirement from formal employment, eligibility for statutory or retirement benefits, political, economic or cultural factors [9,11,12]. In many African countries, the application of a chronological age definition is limited as many elderly people are unaware of their dates of birth thus age groups and social classifications are used while governments apply an age of retirement from formal employment. Although the National Population Commission in Nigeria defines the elderly as those aged 65 years and above and the chronological age of retirement begins at 60 years [9,13], previous studies on the elderly in Nigeria have used 60 years as the minimum for defining people as elderly [14-16]. Following this pattern in this study, we restricted the caregivers to those between the ages of 18 to 59 years who provided care to persons of at least 60 years.

The proportion of the global population aged 60 years and above is increasing rapidly and is projected to double from 11% in 2006 to 22% in 2050 [17,18]. This is partly due to the global increase in life expectancy coupled with the reduction in fertility rates [19-21]. There is thus an increase in the proportion of elderly people with increased pressure on their caregivers such as children and younger siblings [22-24]. The trend of increasing proportions of the elderly population is more marked in developing than in developed countries [20]. This increase is projected to continue so that by the year 2050, people above 60 years of age living in developing countries will be >80% compared with 60% in 2005 [25-27]. Nigeria is reputed to have the highest elderly population in sub-Saharan Africa [18,23]. The proportion of the Nigerian population aged 65 years and above is 3.1% with an elderly dependency ratio of 5.2% according to a 2016 estimation [28]. Also by the year 2025, it is estimated that the elderly population would have grown to 6% and by 2050 the proportion of elderly persons would be 9.9% of the total population [29].

Health care needs increase with age and may cause tension between the older person and their caregivers or others in their social network [30]. Older people are prone to developing disabilities due to the accumulation of health risks across a lifespan of disease, injury and chronic illness which increases the likelihood of dependency and their need for care [25]. In such instances, providing care may prove to be a challenging task [31]. Therefore, the caregivers may experience high levels of stress and burden which may be physical, financial or psychological. Literature abounds on caregiver burden among people who provide care for persons with cognitive impairment such as dementia or Alzheimer´s disease [32-35] and stroke [36,37]. In this context, the term caregiver burden has been used to refer to the many physical, psychological, and socio-economic problems that arise from caring for an individual with a chronic and disabling disease [34]. Kim *et al*. [38] on the other hand defined caregiver burden as *“a multidimensional response to the negative appraisal and perceived stress resulting from taking care of an ill individual”*.

This study was part of an exploration of elder abuse perpetration among adult caregivers of elderly persons and attempts to assess the burden of care among caregivers whose care recipients are not necessarily known to have any cognitive impairment or chronic illness. We aimed to determine the burden of care experienced by caregivers of elderly persons in an informal setting in Nigeria using the modified short version of the Zarit Burden Interview [39]. Different measures of caregiver burden exist, many of which are specific for caregivers of patients with cognitive impairment [40]. The modified short version of the Zarit Burden Interview is a 12-item tool that was developed for screening purposes [39]. This version has been validated and shown to be a reliable tool for assessing the burden of care among informal caregivers of elderly persons [41].

## Methods

**Study design and setting:** a descriptive cross-sectional study was conducted among adult caregivers of elderly persons aged 18 to 59 years in Oyo State, Nigeria between March and May 2016. Oyo State is a southwestern state in Nigeria which has 33 local government areas (LGAs) divided into 12 urban, 9 peri-urban and 12 rural based on the availability of social amenities and the presence of industries in the urban centres or predominance of farmlands in the rural areas. The peri-urban LGAs have a mix of urban and rural characteristics. The study was conducted in two local government areas (LGAs), Afiíjioó a rural LGA and Ibadan South-East which is urban [42,43].

**Sampling and data collection:** a multistage sampling technique was used to select 1,119 caregivers of elderly persons from the selected local government areas. In the first stage, random sampling was done by balloting to select one rural and one urban LGA from the sampling frame of rural and urban LGAs. The second stage involved selecting four (out of twelve) wards from Ibadan South-East (urban) and two (out of ten) wards from Afiíjioó a rural LGA (rural) by balloting using proportional allocation. All the communities in the selected wards were visited. The third stage involved the selection of one caregiver from households with an elderly person that had more than one caregiver in the community by balloting. The sample size was calculated using the formula for comparison of two proportions [44].


$$N=\frac{{\left(Z_{1-\alpha /2}+Z_{1-\beta }\right)}^{2}[P_{1}(1-P_{1})+P_{2}(1-P_{2})]}{{\left(P_{1}-P_{2}\right)}^{2}}$$

Where: N = minimum sample size in each group (rural and urban areas); Z_1-α/2_= standard normal deviate corresponding to the probability of type 1 error (α) at 5% = 1.96; Z_1-β_= standard normal deviate corresponding to the probability of type 2 error (β) of 20%. Power at 80% = 0.84; P_1_and P_2_= proportion of caregivers calculated from a pilot test of the interview guide to assess elder abuse perpetration among adult caregivers in a rural (P_1_= 71.8%) and an urban (P_2_= 24.4%) community. Considering a design effect of 1.5 and a non-response rate of 10%, the minimum sample size calculated was 510 per community. An interviewer-administered, structured questionnaire was used to collect information on sociodemographic characteristics, caregiving arrangements and burden of care experienced by the caregivers.

**Eligibility criteria:** in the context of this study, we defined informal caregivers following Pearlin *et al*. criteria as those who were involved in providing help and assistance to relatives or community members who are unable to provide for themselves [45]. Individuals who were aged between 18 and 59 years who provided care for elderly persons either as paid employees or family members were eligible to participate in the study. The data for this article is part of a study that examined perception and perpetration of elder abuse among informal caregivers. Part of the objectives of the main study was to determine the cultural definition of the elderly at the community level which was done qualitatively. The lowest age mentioned during the qualitative aspect of the main study was 60 years and since this was consistent with the age limit used in previous studies [14-16], we applied 60 years as the age limit for the elderly.

**Measurement of variables:** the level of burden of care experienced was determined using the 12-item Zarit Burden Interview (ZBI) [39]. The modified short version of the Zarit Burden Interview is composed of 12 questions for screening caregivers for the experience of burden of care [39]. The response to each of the questions is graded as “never”, “rarely”, “sometimes”, “quite frequently” and “nearly always” with “never” having a score of 0 while all other responses are scored 1 [39]. The scores are then summed and a total score of 0 implies no experience of burden of care while scores ≥1 are positive for the experience of burden of care. The degree of burden experienced is classified in quartiles with the first quartile being equivalent to mild burden while the last quartile signifies severe burden [39].

The level of dependence for care was measured with the Katz assessment of dependence for Activities of Daily Living (ADL) [46]. This scale has six (6) domains, each scoring a point (1) for independence and 0 for dependence. The least obtainable score is 0 implying total dependence while the highest score is 6 for independence [46]. A score of 6 indicates full function, 4 is moderate functional impairment while scoring 2 or less is classified as having severe functional impairment [46]. To estimate dependence for care in this study, elderly persons with a score of 6 were classified as “independent”, those with a score of 3-5 as “moderately dependent” while those scoring ≤2 were classified as being “fully dependent”. The mental health status of the caregivers was assessed using the 12-item General Health Questionnaire (GHQ-12) [47]. Both the English and Yoruba versions of the GHQ-12 have been validated for use in Nigeria [48,49]. Caregivers who obtained a score of “≥3” were classified as having a poor mental health state [47,50].

Wealth index served as a proxy for socioeconomic status and was determined using the possession of certain household items identified as significant on the Nigerian Demographic and Health Surveys [51]. The items used were type of housing, material for floor finishing, cooking fuel used in the household, source of drinking water, type of toilet facility, ownership of electricity generating set, radio, television, mobile telephone, refrigerator, cable television, air conditioner, electric iron, fan and a means of transportation. Applying Principal Component Analysis (PCA), indicator weights were assigned, and the first factor produced was used to represent the socioeconomic status of the respondents. A weighted frequency distribution of households was obtained and used to generate quintiles classified into the lowest, second, middle, fourth and highest socioeconomic classes.

Data analysis was carried out on IBM SPSS version 22 using descriptive statistics, Chi-square test and logistic regression. Associations between sociodemographic characteristics, caregiving factors and experience of caregiver burden were determined using the Chi-square test. Independent predictors of caregiver burden were determined by logistic regression. Location (independent variable) and other variables such as relationship with the elderly, duration of care giving and mental health status of the caregiver which were significant at bivariate analysis were loaded unto the regression model as co-variates in the analysis. The following variables which have been associated with caregiver burden in other studies [38,40,52,53] were included in the regression model as potential confounders: wealth index, marital status, relationship with the elderly, age and sex. The level of significance was set at 5%.

**Ethical considerations:** participants in the study provided informed consent and confidentiality was maintained by deidentifying the data provided and making it accessible only to the researchers. Ethical approval was obtained from the Oyo State Ministry of Health Ethics Committee (approval number AD/13/479/794).

## Results

**Sociodemographic characteristics:** the mean age of the caregivers interviewed was 38.6 ± 8.7 years with about half (50.2%) aged 40 years and above. There were more females (59.8%) than males (40.2%) and 78.4% of them were married (Table 1). The highest level of education attained by 53.6% of the respondents was secondary education while the commonest occupation was trading (46.5%).

**Table 1:** sociodemographic characteristics of the caregivers

Characteristic	Frequency (%) N=1119
**Age in years (mean = 38.6 ± 8.7)**	
18 to 24	49 (4.4)
25 to 39	508 (45.4)
40 and above	562 (50.2)
**Sex**	
Male	450 (40.2)
Female	669 (59.8)
**Marital status**	
Single	242 (21.6)
Married	877 (78.4)
**Level of education**	
No formal education	56 (5.0)
Completed primary education	212 (18.9)
Completed secondary education	600 (53.6)
Completed tertiary education	251 (22.4)
**Occupation**	
Civil servants	56 (5.0)
Student	57 (5.1)
Teaching	99 (8.8)
Farming	107 (9.6)
Artisans	280 (25.0)
Trading	520 (46.5)
**Wealth index**	
Poorest	257 (23.0)
Poorer	208 (18.6)
Middle	206 (18.4)
Richer	225 (20.1)
Richest	223 (19.9)
**Location**	
Urban	563 (50.3)
Rural	556 (49.7)

**Caregiving arrangements:** about half (52.2%) of the respondents were not the primary caregivers for the elderly but had assistance with the care of the elderly (Table 2). A little more than half (54%) had been caregivers for between one and five years, with the same proportion (54%) caring for their parents while only 2% cared for non-relatives. Most (81%) of the caregivers reported having good relationships with the elders before the need for caregiving.

**Table 2:** details about the caregiving provided to elderly

Characteristic	Frequency (%) N=1119
**Receive assistance with caregiving**	
Yes	535 (47.8)
No	584 (52.2)
**Duration as caregiver**	
< 1 year	76 (6.8)
1 to 5 years	604 (54.0)
6 to 9 years	179 (16.0)
≥ 10 years	78 (16.3)
Not stated	182 (16.3)
**Caregiver’s mental health status**	
Good	255 (22.8)
Poor	864 (77.2)
**Relationship with elder**	
Parent	605 (54.0)
Other relatives	197 (17.6)
Spouse's parent	146 (13.0)
Grandparent	117 (10.5)
Spouse	32 (2.9)
Not a relative	22 (2.0)
**Quality of relationship with elder before caregiving**	
Good	906 (81.0)
Indifferent	167 (14.9)
Poor	46 (4.1)
**Dependence for care by the elderly**	
Independent	898 (80.3)
Dependent	221 (19.7)
**Level of dependence of the elderly**	
Fully independent	898 (80.3)
Moderately dependent	93 (8.3)
Fully dependent	128 (11.4)
**Presence of burden of care**	
Yes	828 (74.0)
No	291 (26.0)
**Level of burden experienced**	
No burden	291 (26.0)
Mild burden	203 (18.1)
Moderate burden	309 (27.6)
Severe burden	316 (28.2)

**Experience of burden of care:** although 80.3% of the elderly persons were reported to be fully independent for activities of daily living (ADL), many (74.0%) of the caregivers experienced burden of care with 28.2% reporting severe burden (Table 2).

**Factors associated with burden of care among caregivers:** the factors found to be significantly associated with the experience of burden of care on bivariate analysis were the wealth index, relationship with the elderly person, the quality of relationship before caregiving began and duration of caregiving (p<0.05). The proportion demonstrating burden of care was highest among those in the poorer wealth quintile, those who cared for their spouses, those who had a poor relationship with the elderly person for whom they provided care and those who had provided care to the elderly for 10 years or more (Table 3).

**Table 3:** odds of experiencing burden of care by caregivers

	Experience of burden of care			Odds (95% CI)		p-value
	Yes	No	Total	Unadjusted	Adjusted	
**Sex**						
Male	328 (72.9)	122 (27.1)	450 (100)	1	na	
Female	500 (74.7)	169 (25.3)	669 (100)	1.10 (0.84-1.44)		
**Age (years)**						
18 to 24	39 (79.6)	10 (20.4)	49 (100)	1	na	
25 to 39	363 (71.5)	145 (28.5)	508 (100)	0.64 (0.31-1.32)		
≥ 40	426 (75.8)	136 (24.2)	562 (100)	0.80 (0.39-1.65)		
**Level of education**						
No formal	39 (69.6)	17 (30.4)	56 (100)	1	na	
Completed primary	160 (75.5)	52 (24.5)	212 (100)	1.34 (0.70-2.57)		
Completed secondary	440 (73.3)	160 (26.7)	600 (100)	1.20 (0.66-2.18)		
Completed tertiary	189 (75.3)	62 (24.7)	251 (100)	1.33 (0.70-2.51)		
**Wealth index**						
Poorest	177 (68.9)	80 (31.1)	257 (100)	1	na	
Poorer	170 (81.7)	38 (18.3)	208 (100)	2.02 (1.30-3.14)	1.84 (1.31-2.98)	0.014
Middle	154 (74.8)	52 (25.2)	206 (100)	1.34 (0.89-2.02)	1.14 (0.72-1.80)	0.571
Richer	170 (75.6)	55 (24.4)	225 (100)	1.40 (0.93-2.09)	1.35 (0.86-2.11)	0.193
Richest	157 (70.4)	66 (29.6)	223 (100)	1.08 (0.73-1.59)	0.83 (0.53-1.31)	0.422
**Marital status**						
Single	164 (67.8)	78 (32.2)	242 (100)	1	1	
Married	664 (75.7)	213 (24.3)	877 (100)	1.48 (1.09-2.02)	0.99 (0.70-1.39)	0.986
**Location**						
Urban	362 (64.3)	201 (35.7)	563 (100)	1		
Rural	466 (83.8)	90 (16.2)	556 (100)	2.88 (2.16-3.82)	10.09 (5.99-17.01)	<0.001*
**Relationship with the elderly**						
Not a relative	14 (63.6)	8 (36.4)	22 (100)	1	1	
Parent	433 (71.6)	172 (28.4)	605 (100)	1.44 (0.59-3.49)	1.56 (0.57-4.27)	0.389
Spouse's parent	122 (83.6)	24 (16.4)	146 (100)	1.91 (1.10-7.68)	2.45 (0.80-7.49)	0.115
Spouse	31 (96.9)	1 (3.1)	32 (100)	17.71 (2.02-155.54)	4.37 (0.42-45.73)	0.219
Grandparent	78 (66.7)	39 (33.3)	117 (100)	1.14 (0.44-2.96)	1.18 (0.40-3.47)	0.758
Other relatives	150 (76.1)	47 (23.9)	197 (100)	1.82 (0.72-4.62)	1.18 (0.41-3.45)	0.749

na: not applicable; * significant

**Predictors of experience of burden of care:** as shown in Table 3 (suite), the adjusted predictors of experience of burden of care were location, duration of caregiving, mental health status of the caregivers and the level of dependence of the elders for ADL. Those who resided in rural areas had 9 times higher odds of experiencing burden of care than those in the urban areas. The odds of experiencing burden of care was about 8 times higher among caregivers with poor mental health status and about 3 times higher among those caring for fully dependent elderly persons.

**Table 3(suite):** odds of experiencing burden of care by caregivers

	Experience of burden of care			Odds (95% CI)		p-value
	Yes	No	Total	Unadjusted	Adjusted	
**Quality of relationship before caregiving**						
Poor	44 (95.7)	2 (4.3)	46 (100)	1	1	
Indifferent	139 (83.2)	28 (16.8)	167 (100)	0.23 (0.05-0.99)	0.34 (0.07-1.71)	0.191
Good	645 (71.2)	261 (28.8)	906 (100)	0.11 (0.03-0.47)	0.56 (0.11-2.92)	0.489
**Duration as caregiver**						
< 1 year	48 (63.2)	28 (36.8)	76 (100)	1	1	
1 to 5 years	447 (74.0)	157 (26.0)	604 (100)	1.66 (1.01-2.74)	2.24 (1.10-4.57)	0.026
6 to 9 years	140 (78.2)	39 (21.8)	179 (100)	2.09 (1.17-3.76)	2.92 (1.24-6.87)	0.014
≥ 10 years				2.67 (1.27-2.50)		
**Receive assistance with care**						
No	411 (70.4)	173 (29.6)	584 (100)	1	1	
Yes	417 (77.9)	118 (22.1)	535 (100)	1.49 (1.14-1.89)	1.15 (0.85-1.55)	0.360
**Mental health status of the caregiver**						
Good	165 (64.7)	90 (35.3)	255 (100)	1	1	
Poor	663 (76.7)	201 (23.3)	864 (100)	1.78 (1.33-2.43)	7.90 (4.60-13.57)	<0.001*
**Dependence for care**						
Independent	632 (70.4)	266 (29.6)	898 (100)	1	1	
Dependent	196 (88.7)	25 (11.3)	221 (100)	3.30 (2.12-5.13)	2.74 (1.68-4.47)	<0.001*
**Care negatively impacts other relationships**						
No	267 (28.4)	674 (71.6)	941 (100)	1	1	
Yes	24 (13.5)	154 (86.5)	178 (100)	2.54 (1.62-4.00)	1.13 (0.67-1.91)	0.658

na: not applicable; * significant

## Discussion

This study assessed the experience of caregiver burden among informal caregivers of elderly persons using the 12-item Zarit Burden Interview (ZBI). The uniqueness of this study is its focus on caregivers of elderly persons who have no known diagnosis of cognitive impairment or other diseases. Previous studies assessing caregiver burden have been among caregivers of people with chronic medical conditions, dementia or other forms of cognitive impairment [54-56]. Comparison of our findings with existing literature is thus limited by the scarcity of studies with a similar participant population. Most of the participants in this study were caregivers for their relatives, which is consistent with the existing literature [4,5,24]. It is expected that younger family members would care for their older relatives and multigenerational households are a common finding in sub-Saharan Africa [34]. Elderly persons whose children are unable to provide care for them or visit regularly have been known to report feeling neglected as documented in existing literature [4,57]. Most of the caregivers had experienced some degree of burden of care with more than one quarter having experienced severe burden of care as measured by the 12-item ZBI.

Female caregivers have been shown to experience a higher degree of caregiver burden in previous studies due to their predisposition to assuming multiple roles in addition to their caregiving roles [33,38,40]. Conversely, some studies reported that women are more exposed to higher caregiving burden which predisposes them to poorer mental and physical health [52,53,58]. Sex was however not significantly associated with the experience of burden of care in our study population. Similar to our findings, a study among caregivers of people with dementia found no significant association between sex and experience of caregiver burden [59].

The proportion who experienced burden of care was highest among those who cared for their spouses followed by those who cared for their spouse´s parents. This is consistent with findings from a previous study where caregivers who were spouses or lived with the care recipients experienced more burden of care [38]. Other studies have also reported a higher burden of care among spouses and children of the care recipient [33,40]. The experience of burden in such instances may be because being related to and living with the care recipient puts more pressure on the caregiver due to longer time spent together as well as familial connections and emotional closeness [38].

Residing in the rural area was highly predictive of experiencing burden of care. This may be related to the fact that many young people migrate out of the rural areas in search of better economic opportunities [29]. The responsibility for the care of the elderly thus falls on the few people remaining in the rural areas putting pressure on them to care both for themselves and their families as well as the elderly persons. With a large proportion of the population living below the poverty level, economic pressures may be a cause of increased burden on the caregivers [29]. This is supported by our study´s finding of people in the poorer wealth category experiencing burden of care compared with other categories.

Caregivers´ mental health status was predictive of experiencing burden of care. Caregivers with poor mental health status had higher odds of experiencing burden of care than those with good mental health status. Perhaps one´s outlook on life influences their relationship and affects their view of the responsibility of caregiving thus influencing their experience of burden of care. This may be further supported by Lin *et al*. finding of caregivers´ mood status as an independent predictor of burden among family and paid caregivers of individuals with dementia [35].

Having an elderly care recipient who is fully dependent for activities of daily living was a positive predictive factor for experiencing burden of care. Kim *et al*. [38] reported that increasing impairment in terms of activities of daily living increases the burden of care for the caregiver because there is a need for higher levels of caregiver engagement.

**Study limitations:** the perspectives presented in this study were those of the caregivers and there may have been some recall or desirability bias associated with the reports. A balance could have been achieved if the opinions of the elderly persons receiving care from the particpants was sought. This was not feasible due to logistic constraints at the time of the study. In addition, the aim of the study was to present the experience of caregivers as this is currently not well reported in extant literature.

## Conclusion

Many of our study participants experienced burden of care as assessed by the 12-item Zarit Burden Interview. The independent predictors of experience of caregiver burden were the location of residence i.e. rural or urban, caregivers´ mental health status and dependence of the elderly person for activities of daily living. Long term intervention to reduce the burden of care among caregivers will include improving the standard of living in the rural areas by ensuring adequate social amenities. This will serve to reduce migration to urban areas thus ensuring there are more people available to provide care for elderly persons in the rural area. Provision of a support system, such as day-care centres for elderly people and home visits will also help to alleviate the burden due to dependence for activities of daily living. Screening programs to identify caregivers of elderly persons experiencing burden of care can be provided at primary health care centres. Caregivers who are found to have poor mental health status may require psychological support, counselling and if necessary, mental health treatment.

### What is known about this topic

- The proportion of elderly persons is increasing globally and this has increased the pressure on those who provide care to the elderly;
- Caregiver burden is recognised among people who care for elderly people with cognitive impairment or other chronic conditions;
- The Zarit Burden Interview is a validated screening tool that can be used to assess caregiver burden.

### What this study adds

- Caregivers of elderly persons within family settings can experience severe burden of care especially when the elderly are dependent for activities of daily living;
- Experience of burden of care is higher among caregivers living in rural than urban areas;
- Caregivers experience of burden may not be negatively influenced by the quality of their relationship with the elderly to whom they provide care.
